# The Incidence of Skin and Soft Tissue Infections in the United States and Associated Healthcare Utilization Between 2010 and 2020

**DOI:** 10.1093/ofid/ofae267

**Published:** 2024-05-07

**Authors:** Venanzio Vella, Dominique Derreumaux, Emmanuel Aris, Michele Pellegrini, Mario Contorni, Michael Scherbakov, Fabio Bagnoli

**Affiliations:** Vaccine Epidemiology–Bacterial, GSK, Siena, Italy; Real World Analytics, GSK, Wavre, Belgium; Real World Analytics, GSK, Wavre, Belgium; Vaccines Clinical Sciences, GSK, Siena, Italy; Early Bacterial Vaccine Program, GSK, Siena, Italy; Global Medical Affairs, GSK, Wavre, Belgium; Infectious Diseases Research Unit, GSK, Siena, Italy

**Keywords:** chronic ulcer, epidemiology, healthcare costs, incidence, skin and soft tissue infections

## Abstract

**Background:**

The number of patients with skin and soft tissue infections (SSTIs) in the United States appeared to be increasing well into the 21st century. However, no recent data have confirmed this trend.

**Methods:**

This retrospective, observational cohort study used claims data over 11 years (2010–2020) from Optum's de-identified Clinformatics Data Mart Database. SSTI episodes, complications, and comorbidities were identified using *International Classification of Diseases* codes. Annual SSTI incidence rates, proportions of recurrent SSTI, SSTI-associated deaths, and total costs were estimated.

**Results:**

During the study period, 5.4 million patients experienced 9.1 million SSTI episodes, with an incidence of 77.5 (95% confidence interval, 77.4–77.5) per 1000 person-years of observation (PYO). Annual incidence did not change significantly over time. Overall incidence (per 1000 PYO) of SSTI episodes in patients without comorbidities was 32.1 (highest incidence was for previous SSTI [113.5]) versus much higher rates if comorbidities were present. Incidence rates (per 1000 PYO) of chronic ulcers increased over time from 11.3 to 18.2 (*P* < .0001) and complicated disease from 3.5 to 6.3 (*P* < .0001). Deaths occurring within 30 days post–SSTI hospitalization rose from 2.6% to 4.6% in 2020. Recurrences occurred in 26.3% of index cases. The mean cost of an SSTI episode was US$3334 (median US$190) and was highest for surgical site infections and chronic ulcers.

**Conclusions:**

The epidemiology of SSTI in the United States is changing and the disease burden is increasing despite stabilization in overall incidence. These data can inform identification of priority populations who could benefit from targeted interventions.

## Teaser

Skin and soft tissue infections in the United States are evolving, with increasing rates of chronic ulcers that frequently develop complications and cause hospitalizations, of which 1% end in death. These data can guide identification of target populations who could benefit most from preventive interventions.

Skin and soft tissue infections (SSTIs) are more common than urinary tract or respiratory infections combined [1]. According to Miller et al, the overall incidence of SSTI between 2005 and 2009 was around 48 per 100 person-years of observation (PYO)—that is, twice that of urinary tract infection and 10 times that of pneumonia [1]. These high rates were confirmed by Casey et al, with 40–45 per 1000 PYO for the same period, and by Ray et al, with 50 per 1000 PYO for the period between 2009 and 2011 [2, 3]. *Staphylococcus aureus* is the most frequently SSTI-associated pathogen, and antibiotic-resistant strains, such as methicillin-resistant *S aureus* (MRSA), complicate treatment and add to treatment costs [3].

Most studies reporting the incidence of SSTI in the United States (US) over the last 3 decades have observed marked increases in SSTIs presenting in the outpatient setting, to emergency rooms (ERs), or requiring hospitalization. Pallin et al [4] reported a 180% increase in ER visits for SSTI between 1993 and 2005; Hersh et al [5] reported a 50% increase in ambulatory visits (outpatient, ER, and physician visits) for SSTI between 1997 and 2005; Edelsberg et al [6] and Kaye et al [7] observed a 29% increase in SSTI hospital admissions between 2000 and 2004 and a 17% increase between 2005 and 2011, respectively; and Lee et al [8] reported a 38% increase in hospitalizations, a 46% increase in ambulatory visits, and a 56% increase in ER visits for SSTI between 2000 and 2012. These trends were thought to be driven by growing antimicrobial resistance including more community-acquired MRSA infections, and infections with resistant forms of less common causes of SSTI such as vancomycin-resistant *Enterococcus* spp, antibiotic-resistant *Pseudomonas aeruginosa*, and extended-spectrum β-lactamase–producing *Escherichia coli* [4–6, 9]. Between 1998 and 2004, 36% of SSTIs caused by *S aureus* in the US were MRSA, increasing to 46% between 2009 and 2011 [3, 10].

In parallel with rising antimicrobial resistance, aging populations with high rates of chronic disease and immune compromise, which are known risk factors for SSTI, could also contribute to increasing infection rates and the development of complicated disease, directly impacting healthcare resource utilization and costs [8, 11, 12].

The most recent US data reporting SSTI incidence stops at 2010 for outpatients [1] and at 2017 for hospital admissions [13], and it is unclear if the rising trends that were recorded in the past have remained the same, have stabilized, or have declined. As microbial resistance patterns, population demographics, and prevalence of chronic diseases such as diabetes mellitus and obesity continue to evolve, updated information on the disease burden due to SSTI in the US is needed to guide health policy and targeted preventive interventions such as potential future vaccination strategies.

To address this data gap, we evaluated the incidence of SSTI episodes, complications, hospitalizations, and mortality using a large healthcare claims database in the US. In addition, we assessed the proportion of patients who developed recurrent SSTI and the healthcare-related costs associated with SSTI.

A plain-language summary of the study is provided in the Supplementary Material.

## METHODS

### Study Design and Data Source

This retrospective, observational cohort study used claims data from 1 January 2010 until 31 December 2020 (31 December 2021 for recurrence data) from Optum's de-identified Clinformatics Data Mart Database (CDM) [14] version 2022 Q2. CDM is an administrative claims database containing medical data, prescription drugs, and outpatient laboratory tests from privately insured individuals with a commercial health plan or Medicare Advantage, distributed around the US. The database currently captures healthcare-related data from >90 million individuals. Most of the analyses were performed on the version of the CDM database containing information about the year of death (CDM DOD view). For specific questions about socioeconomic status, another version of the database (CDM SES view) containing corresponding socioeconomic variables was used.

Subjects of any age were included in the study if they had been continuously enrolled in the database for at least 1 year before entering the study. The study objectives were to estimate the incidence rates of SSTI episodes overall, SSTI with and without complications, hospitalizations, mortality, type of SSTI, the proportion of index SSTI followed by recurrence, and the average cost per SSTI episode.

The study complied with all applicable laws regarding subject privacy. All data were de-identified, were compliant with the Health Insurance Portability and Accountability Act, and only aggregated results were presented; therefore, informed consent and ethics committee approval were not required.

### Outcomes and Case Definitions

SSTI episodes were identified using *International Classification of Diseases*, *Ninth Revision* (*ICD-9*) or *Tenth Revision* (*ICD-10*) codes (see Supplementary material). An SSTI episode started at the date of the first SSTI claim and ended 30 days after the date of the last SSTI claim for that episode. Visits with an SSTI code within the 30-day period were considered part of the same episode. SSTI diagnoses were classified into 4 categories: 1 (most severe) included surgical site infection or infection due to device or graft; 2 included chronic ulcer of skin; 3 included abscess, cellulitis, and erysipelas; and 4 (least severe) included folliculitis, impetigo, furuncle, mastitis, and other. If several different SSTIs were diagnosed during the same SSTI episode, a hierarchy based on clinical judgment was implemented to assign the episode to the most severe category [12]. SSTI-associated procedures (incision and drainage, microbiological laboratory testing, prescription of antibiotics, and prescription of other drugs) occurring within SSTI episodes were captured.

Diagnoses of acute lymphadenitis, myositis, necrotizing fasciitis, gangrene, osteomyelitis, bacteremia, endocarditis, sepsis, and hidradenitis occurring during the SSTI episode were classified as SSTI complications.

Comorbidities associated with an increased risk of SSTI (diabetes mellitus, dermatitis/eczema, obesity, human immunodeficiency virus [HIV], chronic kidney disease including renal dialysis, malignancy [excluding skin], peripheral artery disease, peripheral arteriovenous insufficiency, chronic liver disease, and previous SSTI) were identified using *ICD-9 *and* ICD-10* codes. *ICD* codes used to identify SSTI episodes, procedures, complications, and comorbidities are provided in the Supplementary material.

An index SSTI for the assessment of recurrent episodes was defined if a subject was followed up for at least 1 year after the index SSTI date and if they had been observed for at least 1 year before their index SSTI. Separate biennial cohorts were defined, such that index cases identified during cohort year 1 were followed up for 12 months into cohort year 2 [15]. Patients could be included in >1 biennial cohort with the first SSTI event reported in year 1 of the new cohort considered as a new index case.

SSTI-associated death was defined as the presence of a death record in the CDM database between the admission date and 30 days after discharge in patients hospitalized with a diagnostic-related group (DRG) code for SSTI.

### Statistical Analysis

Frequency tables and proportions were generated for categorical variables. Mean and standard deviation (SD) and 95% confidence interval (CI), median and interquartile range (IQR), and minimum and maximum were generated for cost analyses. Demographic characteristics (age, sex, race/ethnicity, socioeconomic status, and Charlson Comorbidity Index [CCI] score) were assessed at baseline.

Incidence rates and 95% CIs for the entire study period were calculated for outpatient, ER, and inpatient episodes by age group (<5, 5–17, 18–44, 45–64, and ≥65 years), sex, CCI, and presence of comorbidities associated with an increased risk of SSTI. The 95% CIs were calculated for incidence rates according to a binomial distribution. Proportions of SSTI by type and by associated complications were estimated and descriptively analyzed for outpatient, inpatient, and ER SSTI episodes.

Annual incidence rates of SSTI episodes (overall and with complications) and SSTI hospitalization rates were estimated. All incidence rates were estimated per 1000 person-years of observation (PYO) where the total number of PYO for annual incidences of SSTIs was calculated from study entry until the earliest of either the end of continuous enrollment, death, or end of the calendar year. Trends were assessed using a negative binomial regression model.

Deaths occurring during hospitalization and in the first 30 days after discharge for an SSTI episode were reported separately.

The number of index cases and incidence proportion of recurrences was calculated for each biennial cohort over the entire study period.

Medical costs were estimated as the total (US dollars [$]) value of payment to providers/institutions for the SSTI episode. Mean and median SSTI costs were measured in 2020 US dollars adjusted for inflation [16].

## RESULTS

### Study Population

There were approximately 5.4 million patients who had 9.1 million SSTI episodes during the study period; 49.4% of patients were 18–64 years at baseline, 53.3% were female, and 71.5% were White (Table 1). Among patients with income or education data, 24.5% had an income <$40 000, and 79.3% were educated under the level of a bachelor's degree.

**Table 1.** Demographic and Socioeconomic Characteristics of the Study Population

Category	No. of Patients With SSTI	Percentage
Total (CDM DOD view)^a^	5 441 964^a^	
Age group, y		
<5	171 340	3.2
5–17	570 805	10.5
18–44	1 292 865	23.8
45–64	1 395 528	25.6
≥65	2 011 426	37.0
CCI score		
0	3 347 165	61.5
1–2	1 251 697	23.0
3–4	367 189	6.7
≥5	475 913	8.7
Sex		
Female	2 901 069	53.3
Male	2 540 300	46.7
Unknown	595	0.0
Race/ethnicity		
Asian	169 648	3.1
Black	536 996	9.9
Hispanic	548 725	10.1
White	3 889 911	71.5
Unknown	296 684	5.5
Total (CDM SES view)^a^	5 470 117	
Income		
<$40K	1 180 032	21.6
$40K–$49K	370 295	6.8
$50K–$59K	377 760	6.9
$60K–$74K	502 774	9.2
$75K–$99K	725 884	13.3
≥$100K	1 654 847	30.3
Unknown	658 525	12.0
Highest education		
Less than 12th grade	20 740	0.4
High school diploma	1 282 508	23.4
Less than bachelor’s degree	2 857 385	52.2
Bachelor’s degree or higher	1 087 311	19.9
Unknown	222 173	4.1

Abbreviations: CCI, Charlson Comorbidity Index; CDM, Clinformatics Data Mart Database; DOD, date of death; SES, socioeconomic status; SSTI, skin and soft tissue infection.

^a^In analyses using CDM DOD view, patients with recorded date of birth after study end or date of death before the start of enrollment were removed from all analyses. DOD was not available in the CDM SES view, so those patients could not be removed for SES analyses.

### Incidence of SSTI

**Figure 1.**
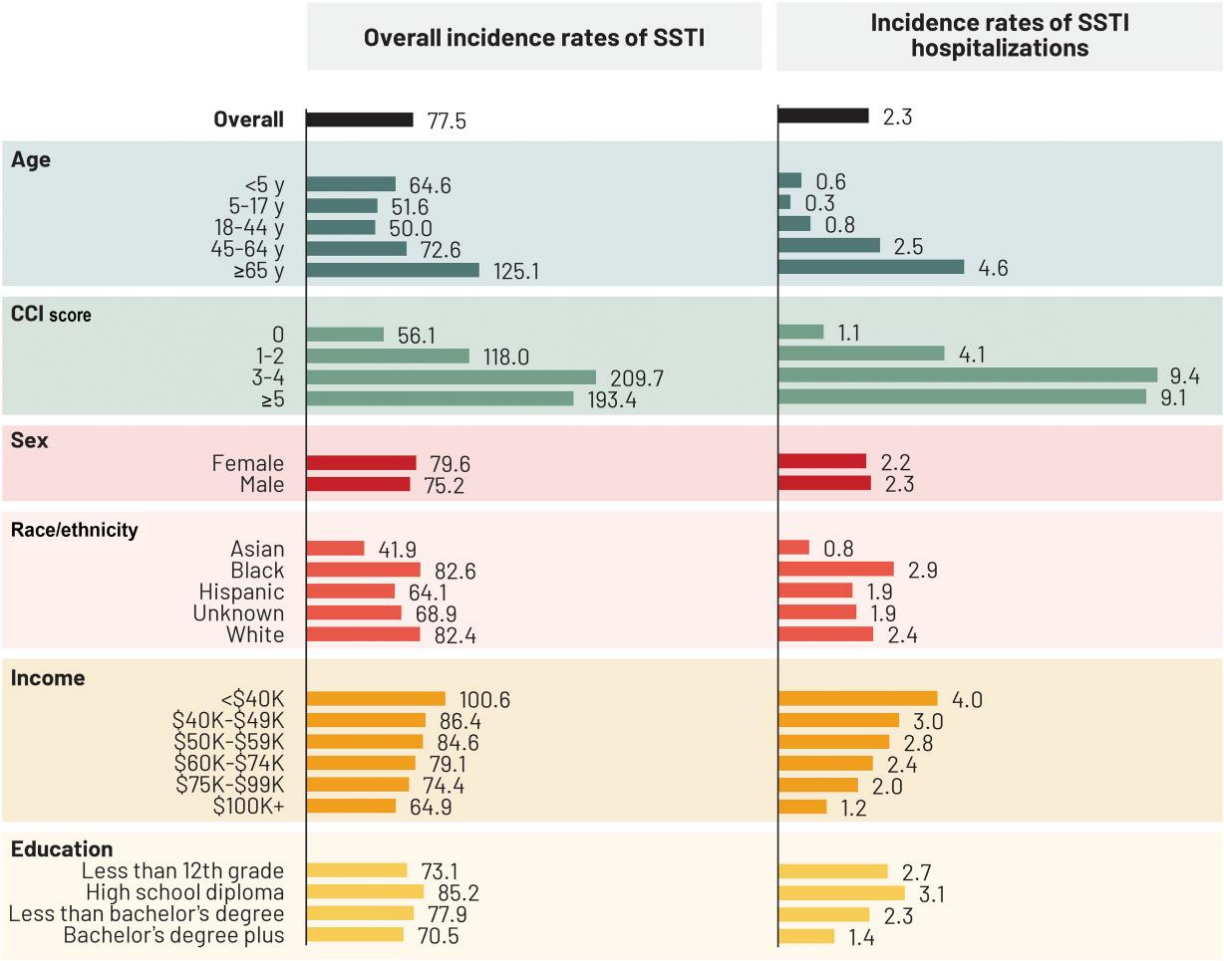
Incidence rates of skin and soft tissue infection (SSTI) and hospitalizations for SSTI per 1000 person-years of observation by demographic and socioeconomic factors. Abbreviations: CCI, Charlson Comorbidity Index; SSTI, skin and soft tissue infection.

The overall incidence of SSTI over the 11-year study period was 77.5 (95% CI, 77.4–77.5) per 1000 PYO (Table 2). Incidence rates were slightly lower for males compared to females (75.2 vs 79.6 per 1000 PYO) and were higher in the age group ≥65 years (125.1 per 1000 PYO). SSTI incidence rates increased from 56.1 per 1000 PYO for a CCI of 0 to approximately 200 per 1000 PYO for a CCI ≥3 (Table 2). The incidence rates decreased from 100.6 per 1000 PYO for incomes <$40 000 to 64.9 per 1000 PYO for incomes ≥$100 000 (Figure 1). A similar relationship between incidence and risk factors was observed for SSTI-associated hospitalizations (Figure 1). Most SSTIs were diagnosed in the outpatient setting (Supplementary Table 1).

**Table 2.** Incidence of Skin and Soft Tissue Infection Overall and by Patient Characteristics

Characteristic	No. of Episodes	Days at Risk	Incidence Rate per 1000 PYO (95% CI)
Age group, y			
<5	204 889	1 158 804 889	64.6 (64.3–64.9)
5–17	764 124	5 409 578 058	51.6 (51.5–51.7)
18–44	1 788 773	13 068 871 265	50.0 (49.9–50.1)
45–64	2 281 425	11 473 940 111	72.6 (72.5–72.7)
≥65	4 090 513	11 940 801 046	125.1 (125.0–125.2)
CCI score			
0	4 988 397	32 498 229 482	56.1 (56.0–56.1)
1–2	2 363 658	7 315 511 341	118.0 (117.9–118.2)
3–4	811 034	1 412 513 137	209.7 (209.3–210.2)
≥5	966 649	1 825 741 409	193.4 (193.0–193.8)
Sex			
Female	4 841 739	22 229 434 397	79.6 (79.5–79.6)
Male	4 286 738	20 818 142 866	75.2 (75.1–75.3)
Unknown	1261	4 418 106	104.3 (98.6–110.2)
Race/ethnicity			
Asian	250 589	2 183 183 368	41.9 (41.8–42.1)
Black	932 320	4 120 465 184	82.6 (82.5–82.8)
Hispanic	898 877	5 125 804 597	64.1 (63.9–64.2)
Unknown	449 278	2 381 866 085	68.9 (68.7–69.1)
White	6 598 674	29 240 676 135	82.4 (82.4–82.5)
Healthcare setting			
Inpatient	264 856	43 051 995 369	2.3 (2.2–2.3)
Emergency room	1 065 444	43 051 995 369	9.0 (9.0–9.1)
Outpatient	7 799 438	43 051 995 369	66.2 (66.1–66.2)
Total	9 129 738	43 051 995 369	77.5 (77.4–77.5)

Abbreviations: CCI, Charlson Comorbidity Index; CI, confidence interval; PYO, person-years of observation.

**Figure 2.**
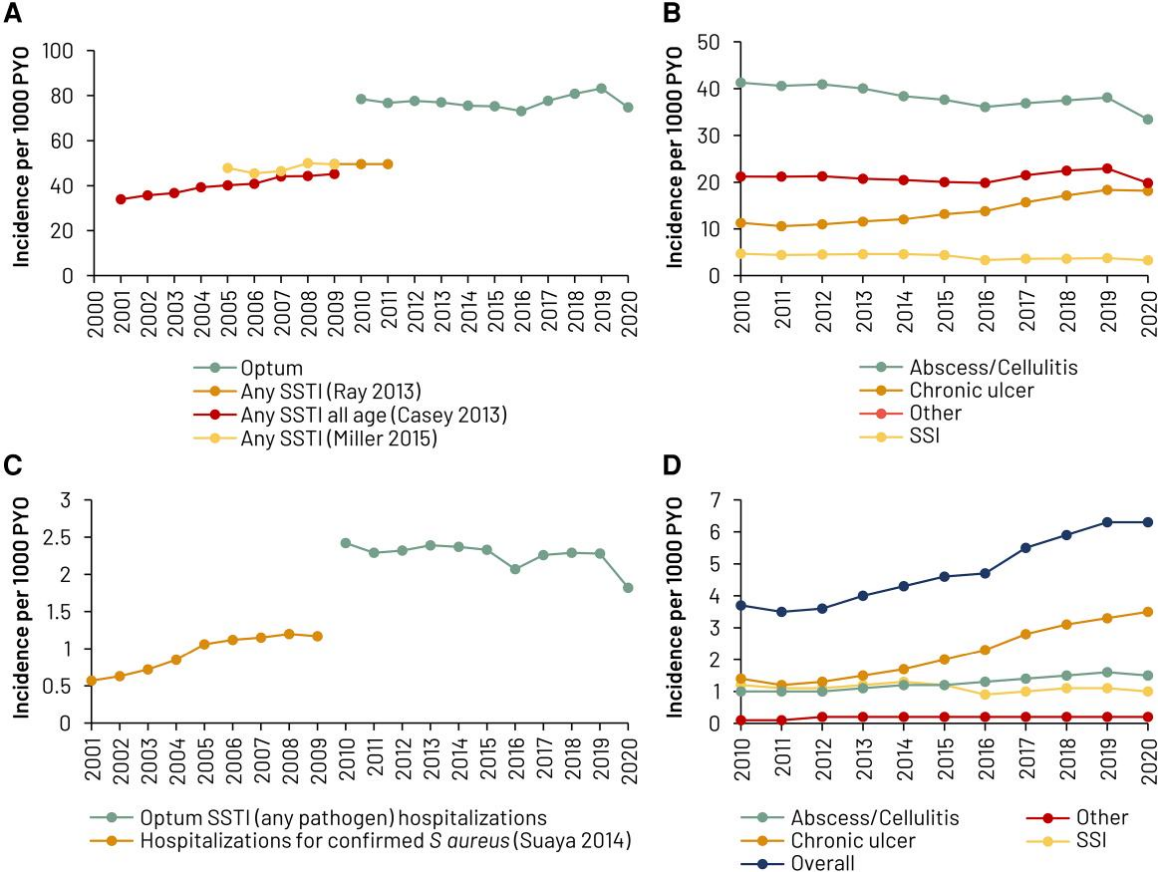
Indirect comparison of the annual incidence rates of skin and soft tissue infection (SSTI) between our study (Optum) and selected previous reports [1–3]. *A*, Any. *B*, By type of SSTI. *C*, Hospitalized SSTI. *D*, SSTI with complications. Other includes folliculitis, impetigo, furuncle, mastitis, and other. Abbreviations: PYO, person-years of observation; SSI, surgical site infection and infection due to device or graft; SSTI, skin and soft tissue infection.

Annual incidence rates ranged between 73.2 and 83.2 per 1000 PYO with a decrease from 2019 to 2020, coinciding with the coronavirus disease 2019 pandemic year (Figure 2). Trend analysis showed a small but nonsignificant increase in the annual rate of SSTI over the study period (*P* = .20). Annual hospitalizations ranged between 2.1 and 2.4 per 1000 PYO except in 2020 (1.8) (*P* = .08).

Abscesses and cellulitis were the most frequent type of SSTI (Figure 2). Over the study period, incidence rates of abscess/cellulitis and surgical site infections decreased somewhat (*P* < .0001 for both), whereas the incidence rate of chronic ulcers increased over time from 11.3 to 18.2 per 1000 PYO (*P* < .0001). Incidence rates of folliculitis/impetigo/furuncle/mastitis/other infections were unchanged over the study period (*P* = .14).

### SSTI With Complications

Rates of SSTI with complications almost doubled during the study period, from a nadir of 3.5 per 1000 PYO in 2011 to 6.3 per 1000 PYO in 2019 and 2020 (*P* < .0001) (Figure 2). This was mainly driven by an increase in complicated chronic ulcers from 1.2 to 3.5 per 1000 PYO. Complications occurred in 6.3% of SSTI episodes overall, most frequently bacteremia/endocarditis/sepsis (4.0%) and osteomyelitis/periostitis/unspecified infection of bone (2.2%) (Table 3). Overall, 27.6% of surgical site infections and 16.1% of chronic ulcers developed complications. The proportions of complications were higher in inpatients (36.1% of SSTI episodes) and patients seen in the ER (20.9%) than in outpatients (3.3%).

**Table 3.** Proportion of Skin and Soft Tissue Infections With Complications

SSTI	No. of Episodes	No. (%) With Complications						
		Any Complication	Lymphadenitis	Myositis	Gangrene	Osteomyelitis^a^	Bacteremia^b^	Hidradenitis
Inpatients								
SSI and device	95 260	49 209 (51.7)	111 (0.12)	1634 (1.72)	6951 (7.3)	17 587 (18.46)	37 733 (39.61)	284 (0.3)
Chronic ulcer^c^	57 847	27 276 (47.2)	47 (0.08)	749 (1.29)	6326 (10.94)	12 261 (21.2)	18 527 (32.03)	146 (0.25)
Abs/cell	110 395	18 999 (17.2)	423 (0.38)	722 (0.65)	1266 (1.15)	2845 (2.58)	14 582 (13.21)	671 (0.61)
Other^d^	1354	154 (11.4)	2 (0.15)	8 (0.59)	11 (0.81)	12 (0.89)	113 (8.35)	24 (1.77)
Total	264 856	95 638 (36.1)	583 (0.22)	3113 (1.18)	14 554 (5.5)	32 705 (12.35)	70 955 (26.79)	1125 (0.42)
Emergency room								
SSI and device	91 712	36 790 (40.1)	38 (0.04)	1937 (2.11)	10 296 (11.23)	16 394 (17.88)	26 817 (29.24)	317 (0.35)
Chronic ulcer^c^	261 250	120 059 (46.0)	72 (0.03)	2014 (0.77)	25 352 (9.7)	47 349 (18.12)	86 886 (33.26)	333 (0.13)
Abs/cell	628 312	62 899 (10.0)	841 (0.13)	1619 (0.26)	3340 (0.53)	8300 (1.32)	50 624 (8.06)	4236 (0.67)
Other^d^	84 170	2361 (2.8)	77 (0.09)	30 (0.04)	139 (0.17)	273 (0.32)	1710 (2.03)	289 (0.34)
Total	1 065 444	222 109 (20.9)	1028 (0.1)	5600 (0.53)	39 127 (3.67)	72 316 (6.79)	166 037 (15.58)	5175 (0.49)
Outpatients								
SSI and device	285 940	44 307 (15.5)	85 (0.03)	1173 (0.41)	6370 (2.23)	17 458 (6.11)	25 735 (9)	834 (0.29)
Chronic ulcer^c^	1 363 391	124 034 (9.1)	94 (0.01)	1144 (0.08)	21 101 (1.55)	60 119 (4.41)	57 357 (4.21)	830 (0.06)
Abs/cell	3 746 627	70 493 (1.9)	2571 (0.07)	1213 (0.03)	4560 (0.12)	18 655 (0.5)	34 528 (0.92)	13 352 (0.36)
Other^d^	2 403 480	18 695 (0.8)	1213 (0.05)	156 (0.01)	1225 (0.05)	2906 (0.12)	6113 (0.25)	7668 (0.32)
Total	7 799 438	257 529 (3.3)	3963 (0.05)	3686 (0.05)	33 256 (0.43)	99 138 (1.27)	123 733 (1.59)	22 684 (0.29)
Overall								
SSI and device	472 912	130 306 (27.6)	234 (0.05)	4744 (1.0)	23 617 (4.99)	51 439 (10.88)	90 285 (19.09)	1435 (0.3)
Chronic ulcer^c^	1 682 488	271 369 (16.1)	213 (0.01)	3907 (0.23)	52 779 (3.14)	119 729 (7.12)	162 770 (9.67)	1309 (0.08)
Abs/cell	4 485 334	152 391 (3.4)	3835 (0.09)	3554 (0.08)	9166 (0.2)	29 800 (0.66)	99 734 (2.22)	18 259 (0.41)
Other^d^	2 489 004	21 210 (0.9)	1292 (0.05)	194 (0.01)	1375 (0.06)	3191 (0.13)	7936 (0.32)	7981 (0.32)
Total	9 129 738	575 276 (6.3)	5574 (0.06)	12 399 (0.14)	86 937 (0.95)	204 159 (2.24)	360 725 (3.95)	28 984 (0.32)

Abbreviations: Abs/cell, abscess, cellulitis, and erysipelas; SSI, surgical site infection and infection due to device or graft; SSTI, skin and soft tissue infection.

^a^Osteomyelitis includes periostitis and unspecified infection of bone.

^b^Bacteremia includes endocarditis and sepsis.

^c^Chronic ulcer of skin.

^d^Other includes folliculitis, impetigo, furuncle, mastitis, and other.

### Comorbidities

Incidence rates of SSTI were higher in patients with at least 1 comorbidity, being highest in patients with a past history of SSTI (281.4 per 1000 PYO), followed by concomitant peripheral artery and arteriovenous disease (222.0 per 1000 PYO), chronic kidney disease (204.3 per 1000 PYO), diabetes mellitus (164.8 per 1000 PYO), malignant neoplasm (161.2 per 1000 PYO), and chronic liver disease (145.7 per 1000 PYO) (Table 4). Similar trends were observed across inpatient, ER, and outpatient settings with multiple comorbidities (Supplementary Table 1).

**Table 4.** Incidence of Skin and Soft Tissue Infection Among Patients With 1 or More Comorbidities

Comorbidity^a^	No. of Patients With This Comorbidity	No. of Episodes	Days at Risk	Incidence Rate per 1000 PYO (95% CI)
Diabetes mellitus	5 694 758	2 960 804	6 564 122 951	164.8 (164.6–164.9)
Dermatitis/eczema	8 167 223	3 004 221	8 900 163 946	123.3 (123.2–123.4)
Obesity	5 266 650	2 090 043	5 591 976 083	136.5 (136.3–136.7)
Previous SSTI	2 239 664	1 901 456	2 468 083 750	281.4 (281–281.8)
HIV/AIDS	100 809	36 990	99 673 243	135.6 (134.2–136.94)
Chronic kidney disease	2 804 218	1 661 349	2 970 818 396	204.3 (204.0–204.6)
Malignant neoplasm	2 321 448	1 011 931	2 292 468 893	161.2 (160.9–161.5)
Peripheral artery and arteriovenous disease	3 485 422	2 356 903	3 878 673 878	222.0 (221.7–222.2)
Chronic liver disease	2 454 993	946 469	2 372 210 492	145.7 (145.4–146.0)

Abbreviations: CI, confidence interval; HIV, human immunodeficiency virus; PYO, person-years of observation; SSTI, skin and soft tissue infection.

^a^Patients with 1 of the listed comorbidities could also have any of the other 8 comorbidities, for example, diabetes only or diabetes associated with 1 or several other comorbidities (eg, dermatitis/eczema).

The incidence rate of SSTI in patients without recorded comorbid conditions was 32.1 per 1000 PYO, while the incidence of SSTI associated with a given individual comorbidity, without any other coexisting comorbidities, varied between 46.6 and 113.5 per 1000 PYO (Table 5). The pattern of SSTI incidence in patients with a single comorbidity was similar to that for those with at least 1 comorbidity, except for dermatitis.

**Table 5.** Incidence of Skin and Soft Tissue Infection Among Patients With No Comorbidity During the Whole Study, or With 1 Comorbidity Already Present at Baseline and With No Additional Ones Recorded During the Study

Single Comorbidity^a^	No. of Patients	No. of Episodes	Days at Risk	Incidence Rate per 1000 PYO (95% CI)
Diabetes	952 099	111 494	752 724 732	54.1 (53.8–54.4)
Dermatitis/eczema	1 994 127	347 403	1 874 780 722	67.7 (67.5–67.9)
Obesity	758 056	76 520	579 567 134	48.2 (47.9–48.6)
Previous SSTI	738 360	188 116	605 506 169	113.5 (113.0–114.0)
HIV/AIDS	26 791	3094	19 623 875	57.6 (55.6–59.7)
Chronic kidney disease	128 273	15 922	103 021 466	56.5 (55.6–57.3)
Malignant neoplasm	238 465	27 773	187 785 364	54.0 (53.4–54.7)
Peripheral artery and arteriovenous disease	202 451	38 035	169 874 279	81.8 (81.0–82.6)
Chronic liver disease	180 582	16 622	130 237 199	46.6 (45.9–47.3)
No comorbidity	21 079 634	1 594 137	18 150 104 383	32.1 (32.0–32.1)

Abbreviations: CI, confidence interval; HIV, human immunodeficiency virus; PYO, person-years of observation; SSTI, skin and soft tissue infection.

^a^Patients with a single comorbidity among the listed comorbidities (eg, diabetes only).

### Recurrent SSTI

**Figure 3.**
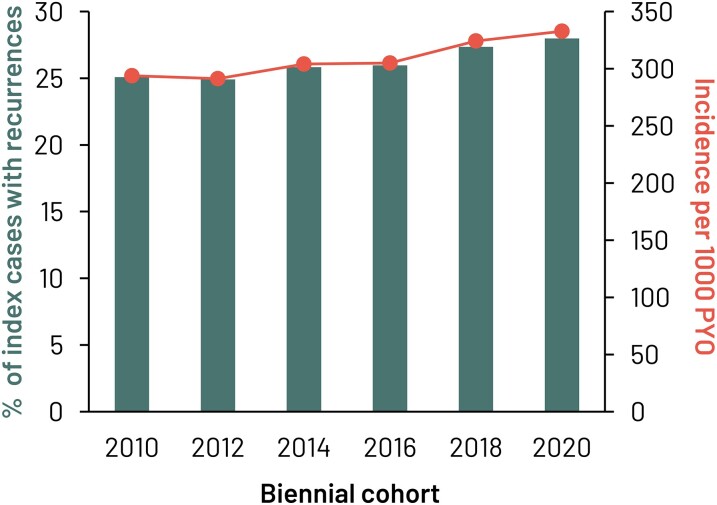
Percentage of index skin and soft tissue infections (SSTIs) with recurrence and incidence of recurrent SSTI. Abbreviation: PYO, person-years of observation.

In the biennial cohorts, there were 2 977 301 index SSTI cases of which 783 963 (26.3%) developed a recurrent SSTI episode. The percentage of index cases with recurrence varied from 24.9% in the 2012–2013 biennial cohort to 28.0% in the 2020–2021 cohort (Figure 3). The overall incidence of recurrences was 310.4 per 1000 PYO, ranging from 291.3 to 332.8 per 1000 PYO annually (Figure 3). Risk factors for recurrences resembled those for all SSTIs, with increasing recurrences in older age groups, and in patients with higher CCI score and lower level of education (Supplementary Figure 1).

### Mortality

**Figure 4.**
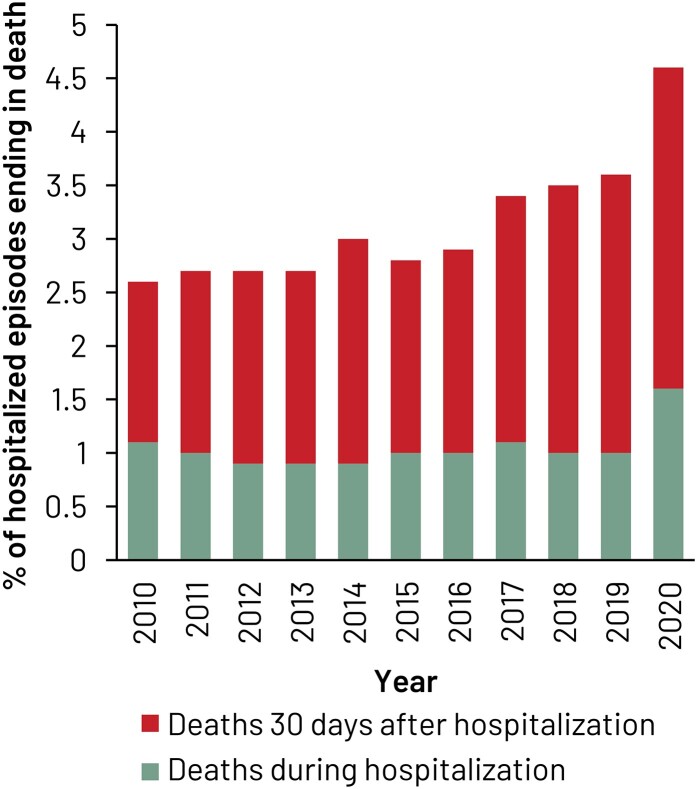
Percentage of skin and soft tissue infection (SSTI) episodes ending in death during or within 30 d after hospitalization. The total length of bars is related to the mortality that occurred between admission up to 30 days after discharge.

Deaths occurring between admission and within 30 days after discharge rose from 2.6% in 2010 to 3.6% in 2019 (4.6% in the 2020 severe acute respiratory syndrome coronavirus 2 [SARS–CoV–2] pandemic year), whereas deaths during SSTI hospitalization changed little over the study period until 2019 (0.9%–1.1%), except for an increase in the 2020 pandemic year to 1.6% (Figure 4).

### Costs Associated With SSTI

The range of healthcare costs associated with SSTI varied widely and is reflected in markedly different mean and median estimates. The mean cost of an SSTI episode during the study period was $3334 (SD, $20 030) and the median cost was $190 (IQR, $96–$510) (Supplementary Table 2). Mean and median costs increased over the study period. Costs tended to be higher in the age groups 45–64 and ≥65 years, males, surgical site infections, and chronic ulcers than for other SSTI types (Supplementary Table 2, Supplementary Figure 2).

The mean and median costs for a hospitalized SSTI episode were $36 515 (SD, $58 567) and $20 071 (IQR, $13 905–$35 541), respectively, versus $467 (SD, $2501) and $146 (IQR, $91–$297), respectively, for an outpatient episode. The distribution of costs among subpopulations by age, sex, and comorbidity was similar in each setting (data not shown).

## DISCUSSION

This study provides updated estimates of the incidence of SSTI in the US. The annual incidence rate of all SSTI episodes between 2010 and 2020 varied between 73.2 and 83.2 per 1000 PYO, which is higher than previously reported in the literature for the US (Figure 2) [1–3], but similar to the 72.9 per 1000 population reported Canada in 2008 [17]. The higher incidence observed in our study might be related to differences in CDM’s population socioeconomic structure, which only includes relatively wealthy individuals with commercial or Medicare Advantage health insurance, or differences in access to services, age groups, or reasons linked to study design and case definition. Hospitalizations for SSTI as primary cause were relatively stable over the study period and were in line with previously published estimates [1]. Hospitalization rates were close to twice the published rate of *S aureus* SSTI hospitalization (Figure 2), which is in line with the hypothesis that about half of SSTIs are caused by *S aureus* [9, 10].

The proportional distribution by type of SSTI leading to hospitalization was in line with the literature, except for chronic ulcers [1, 9]. Approximately 21.8% (57 847/264 856) of inpatient cases of SSTI with complications were attributed to chronic skin ulcers (Table 3), which was higher compared to 10% reported by Miller et al [1]. The incidence rate of any SSTI with complication climbed from 3.5 to 6.3 per 1000 PYO, an increase of approximately 80%, over the study period. Surgical site infections and chronic ulcers were both associated with high rates of complicated disease (27.6% and 16.1%, respectively), the most frequent being bacteremia, osteomyelitis, and gangrene.

Our study confirms the risk factors reported in previous studies that are associated with SSTI [11, 12]. However, while most studies measured such relationship in terms of odds ratios, our study complements these data by quantifying SSTI incidence rates by risk factor. Providing incidence by risk factor brings added value for health technology assessments because it allows quantification of the burden of disease and the identification of potential target populations who could benefit most from preventive interventions.

Intrahospital mortality for SSTI was around 1%. However, mortality until 30 days after discharge for SSTI was higher and increased steadily during the study period to 3.6% by 2019. The 2020 year was characterized by fewer SSTI episodes overall, fewer hospitalizations for SSTI, and higher in-hospital and postdischarge mortality, which is consistent with both reduced access to healthcare during the SARS-CoV-2 pandemic and an increase in complicated SSTI, which is in line with higher mortality. The fact that these deaths were assigned to the DRG used for SSTI strengthens the hypothesis that this higher mortality was directly linked to SSTI and was not simply related to the overall mortality due to COVID-19 during the pandemic.

Approximately one-quarter of the index cases were affected by recurrences, with more recurrences in older patients, those with comorbidities, and patients with low income, which is line with the literature [15, 18–21].

Strengths of the study include the large sample size, the extended study period using contemporary data that provide up-to-date quantifiable estimates of the SSTI disease burden, and the comparison with previous studies on the incidence of SSTI in the US. As for all claims-based studies, the data might not be representative of the entire US population, and clinical information is lacking, which does not allow confirmation of the diagnosis or assessment of severity. The study data, relying on 2 datasets, might be subjected to biases associated with observational and retrospective datasets and is inherently biased to individuals with health insurance. For the assessment of recurrence, individuals were required to have been in the database for 1 year before the index SSTI and followed up for at least 1 year. This will result in some data loss. The mortality rate accounts for deaths occurring from hospital admission to 30 days postdischarge, encompassing hospitalized patients only. Moreover, the mortality rate represents a crude estimate, as demonstrated by the higher mortality observed in 2020, likely attributable to the direct and indirect effects of the COVID-19 pandemic. Another potential limitation is that prescription claims are not directly linked to diagnoses and there may be a risk of misclassification when attributing antibiotic prescriptions to SSTI. Hospitalization costs were based on episodes with DRG values; however, the DRG was missing for >20% of hospitalizations, potentially biasing the overall calculation and the estimation of the mean/median costs.

This retrospective study confirms that SSTIs are far from being on the decline and still cause a substantial disease burden with high rates of complications, high associated healthcare costs, and 1% mortality during hospitalizations. While we did not observe an increase in the incidence rate over the 11-year study period, there was consistent evidence that the epidemiology of SSTI is changing in the US, possibly in response to an aging population, increasing prevalence of chronic diseases known to be risk factors for SSTI, and community-acquired multidrug-resistant infections. This change is characterized by an increasing proportion of chronic ulcers, complicated disease and deaths occurring posthospitalization, and increasing associated healthcare costs. Our study also assessed mortality between the date of admission and 30 days after discharge. Mortality during this period was relatively stable until 2013 but gradually increased between 2017 and 2019 and reached a peak in 2020. These data, that to the best of the authors’ knowledge are not available in the literature, suggests that mortality attributed to SSTI may be higher than previously supposed.

These data are relevant for clinicians and for policy makers. By disaggregating SSTI incidence and hospitalization by age, sex, and key risk factors, this study provides critical parameters for the estimation of the burden of disease, for construction of baseline assumptions for clinical studies aimed at preventing or treating SSTI, and for identification of priority populations who could benefit from targeted interventions. In this context, our study provides the incidence of SSTI associated with a given comorbidity (which might coexist with other competing comorbidities), the incidence associated with each individual comorbidity with no other comorbidities being present, the incidence when there are no comorbidities, and the incidence when multiple comorbidities are present. This information can help policy makers to estimate the SSTI incidence that is attributable to individual comorbidities.

## Supplementary Material

ofae267_Supplementary_Data

## Supplementary Data

Supplementary materials are available at *Open Forum Infectious Diseases* online. Consisting of data provided by the authors to benefit the reader, the posted materials are not copyedited and are the sole responsibility of the authors, so questions or comments should be addressed to the corresponding author.

## Notes

***Acknowledgments.*** The authors thank Amit Adlak and Pratyk Gomez for their programming work on this study. The authors also thank Business & Decision Life Sciences Medical Communication Service Center for editorial assistance and manuscript coordination, on behalf of GSK. Dr Joanne Wolter (independent, on behalf of GSK) provided writing support.

***Author contributions.*** V. V., E. A., and D. D. designed the study and the analytical strategy. D. D. was responsible for data extraction and analytical outputs. V. V., E. A., and D. D. interpreted the results. V. V. and D. D. drafted the manuscript. All authors were involved in the interpretation of the results, the development of this article, and in revising each draft. All authors had full access to the data and gave final approval before submission.

***Patient consent.*** The study complied with all applicable laws regarding subject privacy. All data were de-identified and were compliant with the Health Insurance Portability and Accountability Act, and only aggregated results were presented; therefore, informed consent and ethics committee approval were not required.

***Data availability.*** The data that support the findings of this study are available from Optum, but restrictions apply to the availability of these data, which were used under license for the current study and so are not publicly available. Data are, however, available from the authors upon reasonable request and with permission of Optum.

***Disclaimer.*** The funder was involved in all stages of study conduct, including analysis of the data. GlaxoSmithKline Biologicals SA also took in charge all costs associated with the development and publication of this manuscript.

***Financial support*.** This work was supported by GlaxoSmithKline Biologicals SA (study identifier: VEO-000423; PLWID: B15390).
