# Supplementary material for: The Incidence of Skin and Soft Tissue Infections in the United States and Associated Healthcare Utilization Between 2010 and 2020
Source: Open Forum Infect Dis. 2024 May 7;11(6):ofae267. doi: 10.1093/ofid/ofae267 (PMC11146672; doi:10.1093/ofid/ofae267)
Supplement: ofae267_Supplementary_Data [file ofae267_supplementary_data.zip › EPI_OPTUM db study_supplemental_FINAL.docx]

**The incidence of skin and soft tissue infections in the United States and associated health care utilization between 2010 and 2020**

Venanzio Vella ^1^, Dominique Derreumaux^2^, Emmanuel Aris^2^, Michele Pellegrini^3^, Mario Contorni^4^, Michael Scherbakov^5^, Fabio Bagnoli^6^

1. Vaccine Epidemiology – Bacterial, GSK, Siena, Italy
2. Real World Analytics, GSK, Wavre, Belgium
3. Vaccines Clinical Sciences, GSK, Siena, Italy
4. Early Bacterial Vaccine Program, GSK, Siena, Italy
5. Global Medical Affairs, GSK, Wavre, Belgium
6. Infectious Diseases Research Unit, GSK, Siena, Italy

**SUPPLEMENTAL MATERIAL**

**Plain Language Statement**

*What is the context?*

- Skin and soft tissue infections (SSTI) are very common, and studies conducted previously in the US showed that the incidence of these infections was increasing. These data are up to a decade old, and it is not known if the rate of SSTI is still increasing in the US.

*What is new?*

- Our study evaluates the rate of SSTI in the US, which appears to have stabilized. However, the SSTI profile has been changing, with more patients developing chronic ulcers than before, leading to higher hospitalizations and mortality than previously reported.
- To the best of the authors’ knowledge, this is the first time that the incidence of SSTI has been measured among patients with a single specified comorbidity (e.g., diabetes only) and those with multiple comorbidities (e.g., diabetes and chronic kidney disease), enabling to isolate the attributable incidence of SSTI according to specific comorbidities.

*What is the impact?*

- This study provides up-to-date information on the importance of SSTI in the US, showing that the incidence of SSTI is not declining, contrary to previous assumptions.
- The study helps to identify people at risk of SSTI and to estimate the benefit from preventive interventions to reduce their risk.

**International Classification of Disease (ICD) codes list for skin and soft tissue infections (SSTI)**

| **SSTI type** | **SSTI Diagnosis** | **ICD-9 code** | **ICD-10 code** |
| --- | --- | --- | --- |
| 1 | Surgical site infection | 674.30-.34 , 998.51-.59 | K68.11, O86.0x , O90.2 , T81.4x |
| 1 | Non-healing surgical wound | 998.83 | T81.89XA |
| 1 | Infection due to device or graft | 996.6x | T85.79XA |
| 2 | Chronic ulcer of skin | 707.00-707.09 (decubitus ulcer) | L89.x |
| 1 |  | 707.10-707.19 (ulcer of lower limb, except decubitus ulcer) | L97.x |
| 3 | Abscess | 682xx (including abscess with cellulitis) | L02 with extensions: .01 ,.09 ,.11 ,.211- ,.219 ,.411-419 ,.511-.519 ,.611-.619 ,.811 ,.818 ,.91 |
| 3 | Cellulitis | 681.x | L03 xx |
| 3 | Erysipelas | 035 | A46 |
| 4 | Carbuncle or furuncle | 680.x | L02, with extensions:.02,.03,.12,.13,.221-.239,.421-.439,.521-.539,33,.,.621-.639,.821-838,.92,.93 |
| 4 | Impetigo | 684 | L01.x |
| 4 | Dermatitis infectiosa eczematoides | 690.8 | L30.3 |
| 4 | Folliculitis | 704.8 | L66.3, L73.8 |
| 4 | Mastitis | 611.0x, 675.00-675.14 | N61x, O91.011-O91.12 |
|  |  |  |  |
| 4 | Unspecified local infection of skin or subcutaneous tissue and Superficial injury with mention of infection | 686.x, 910.1, 910.3, 910.5, 910.7, 910.9, 911.1, 911.3, 911.5, 911.7, 911.9, 912.1, 912.3, 912.5, 912.7, 912.9, 913.1, 913.3, 913.5, 913.7, 913.9, 914.1, 914.3, 914.5, 914.7, 914.9, 915.1, 915.3, 915.5, 915.7, 915.9, 916.1, 916.3, 916.5, 916,7, 916.9,  917.1, 917.3, 917.5, 917.7, 917.9, 919.1, 919.3, 919.5, 919.7, 919.9 | L080, L08.89, L089, L88, L980,  S0090XA, S0092XA, S0095XA, S0096XA, S1090XA, S1091XA, S1092XA, S1095XA, S1096XA, S2090XA, S2091XA, S2092XA, S2095XA, S2096XA, S40219A, S40229A, S40259A, S40269A, S40819A, S40829A, S40859A, S40869A, S40919A, S40929A, S50319A, S50329A, S50359A, S50369A, S50819A, S50829A, S50859A, S50869A, S50909A, S50919A, S60319A, S60329A, S60359A, S60369A, S60419A, S60429A, S60459A, S60469A, S60519A, S60529A, S60559A, S60569A, S60819A, S60829A, S60859A, S60869A, S60919A, S60929A, S60939A, S60949A, S70219A, S70229A, S70269A, S70319A, S70329A, S70369A, S70919A, S70929A, S80219A, S80229A, S80269A, S80819A, S80829A, S80869A, S80919A, S80929A, S90413A, S90416A, S90423A, S90426A, S90453A, S90456A, S90463A, S90466A, S90519A, S90529A, S90569A, S90819A, S90829A, S90859A |

1: surgical site infection & infection due to device or graft; 2: chronic ulcer of skin; 3: abscess, cellulitis & /erysipelas; 4: folliculitis, impetigo, furuncle, mastitis & other

**International Classification of Disease (ICD) codes for complications**

| **Diagnosis** | **ICD-9 code** | **ICD-10 code** |
| --- | --- | --- |
| Acute lymphadenitis | 683 | L04.9 |
| Myositis | 728.0 | M60 |
| Necrotising fasciitis | 728.86 | M72.6 |
| Gangrene | 785.4, 040.0x | I96 |
| Osteomyelitis | 730X | M86x |
| Bacteraemia | 790.7 | R78.81 |
| Endocarditis | 421.0, 421.1, 421.2, 421.9 | I33x |
| Sepsis | 038.x, 995.91, 995.92,785.52 | A40x, A41x, R65.20, R65.21 |
| Hidradenitis | 705.83 | L73.2 |

**International Classification of Disease (ICD) codes for comorbidities**

| **Diagnosis** | **ICD-9 code** | **ICD-10 code** |
| --- | --- | --- |
| Any diabetes | 249.x, 250.x | E08.x,09.x, E10.x, E11.x, E12.x, E13.x |
| Chronic kidney disease | 585.x, 403.x | N18.x, I12.x |
|  | 99656, 99668, 99673, | T85.691, T8571XA, |
|  | v4511,4512, v562, v563.x | Z99.2, Z911.5,490.2, Z493.x |
| HIV/AIDS | 42 | B20 |
| Malignant neoplasm | 140.x-172.x, 174.x-195.8, | C00.x-C26.x, C30.x-C34.x, |
|  | 200.x-208.x, 238.6 | C37.x-C41.x, C43.x, C45.x-C58.x, |
|  |  | C60.x-C76.x, C81.x-C85.x, C88.x, C90.x-C97.x |
| Obesity and overweight (body mass index ≥30) | 278.00, 278.01, 278.02, | E661, E662 E668, E669 |
|  | V85.3x, V85.4x, | Z68.3 Z68.4 |
| Peripheral artery and arteriovenous disease including | 440.x, 443.9, 459.x | I70.x, I73.9, I99.x |
| Chronic liver disease | 571.x, 572.x, 573.x | K70.x to K76.x |
| Dermatitis and eczema | 690.x to 695.x | L20x-L30x |

HIV/AIDS: human immunodeficiency virus/acquired immunodeficiency syndrome.

**Procedure code list: 1- Microbiological laboratory testing codes**

| **Current Procedural Terminology Code** | **Description** |  |
| --- | --- | --- |
| 87081 | Culture, presumptive, pathogenic organisms, screening only | Microbiology screening |
| 87084 | Culture, presumptive, pathogenic organisms, screening only; with colony estimation from density chart | Microbiology screening |
| 87252 | Virus isolation; tissue culture inoculation, observation, and presumptive identification by cytopathic effect | Viral Tissue Culture |
| 87253 | Virus isolation; tissue culture, additional studies or definitive identification (eg, hemabsorption, neutralization, immunofluorescence stain), each isolate | Viral Tissue Culture |

**Incision & drainage**

| **Procedure** | **Code** |
| --- | --- |
| Current Procedural Terminology | 10060 ,10061, 10160 |
| Healthcare Common Procedure Coding System | D7510 , D7511, D7520, D7521 |
| ICD-9 procedure | 49.01,86.01 , 86.04 |
| ICD-10 procedure coding system | 0H9BXZZ, 0H9CXZZ, 0H9DXZZ, 0H9EXZZ, 0H9FXZZ, 0H9GXZZ, 0H9HXZZ, 0H9JXZZ, 0H9KXZZ, 0H90XZZ, 0H91XZZ, 0H94XZZ, 0H95XZZ, 0H96XZZ , 0H97XZZ, 0H98XZZ, 0H9AXZZ, 0H9LXZZ, 0H9MXZZ, 0H9NXZZ, 0H9QXZZ, 0H9RXZZ, 0J903ZZ, 0J913ZZ, 0J943ZZ, 0J953ZZ, 0J963ZZ, 0J973ZZ, 0J983ZZ, 0J993ZZ, 0J9B3ZZ, 0J9C3ZZ, 0J9D3ZZ, 0J9F3ZZ, 0J9G3ZZ, 0J9H3ZZ, 0J9J3ZZ, 0J9K3ZZ, 0J9L3ZZ , 0J9M3ZZ , 0J9N3ZZ , 0J9P3ZZ , 0J9Q3ZZ , 0J9R3ZZ , 0W903ZZ , 0W9K3ZZ , 0W9L3ZZ , 0W9M3ZZ |

ICD: International Classification of Diseases (versions 9 and 10)

Table S1 Incidence (per 1000 person-years of observation) of skin and soft tissue infections (SSTI) in patients with one or more than one comorbidity recorded during the baseline period or during the study, by setting

|  | **Multiple comorbidities*** | **N episodes** | **N days at risk** | **Incidence rate for 1000 persons-year** | **95% CI -LL** | **95% CI -UL** |
| --- | --- | --- | --- | --- | --- | --- |
| **Inpatients** | Diabetes | 126,863 | 6,564,122,951 | 7.1 | 7.0 | 7.1 |
|  | Dermatitis/eczema | 79,688 | 8,900,163,946 | 3.3 | 3.3 | 3.3 |
|  | Obesity/overweight | 96,245 | 5,591,976,083 | 6.3 | 6.3 | 6.3 |
|  | Previous SSTI | 69,469 | 2,468,083,750 | 10.3 | 10.2 | 107.8 |
|  | HIV/AIDS | 1,640 | 99,673,243 | 6.0 | 5.7 | 6.3 |
|  | Chronic kidney disease | 79,163 | 2,970,818,396 | 9.7 | 9.7 | 9.8 |
|  | Malignant neoplasm | 47,826 | 2,292,468,893 | 7.6 | 7.6 | 7.7 |
|  | Peripheral artery and arteriovenous disease | 106,405 | 3,878,673,878 | 10.0 | 10.0 | 10.1 |
|  | Chronic liver disease | 45,019 | 2,372,210,492 | 6.9 | 6.9 | 7.0 |
|  |  |  |  |  |  |  |
| **ER** | Diabetes | 439,276 | 6,564,122,951 | 24.4 | 24.4 | 24.5 |
|  | Dermatitis/eczema | 299,879 | 8,900,163,946 | 12.3 | 12.3 | 12.4 |
|  | Obesity/overweight | 308,561 | 5,591,976,083 | 20.2 | 20.1 | 20.2 |
|  | Previous SSTI | 268,812 | 3,006,913,019 | 32.7 | 32.5 | 32.8 |
|  | HIV/AIDS | 6,701 | 99,673,243 | 24.6 | 24.0 | 25.2 |
|  | Chronic kidney disease | 266,256 | 2,970,818,396 | 32.7 | 32.6 | 32.9 |
|  | Malignant neoplasm | 137,584 | 2,292,468,893 | 21.9 | 21.1 | 22.0 |
|  | Peripheral artery and arteriovenous disease | 335,402 | 3,878,673,878 | 31.6 | 31.5 | 31.7 |
|  | Chronic liver disease | 147,327 | 2,372,210,492 | 22.7 | 22.6 | 22.8 |
|  |  |  |  |  |  |  |
| **Outpatient** | Diabetes | 2,394,665 | 6,564,122,951 | 133.3 | 133.1 | 110.2 |
|  | Dermatitis/eczema | 2,624,654 | 8,900,163,946 | 107.7 | 107.6 | 161.3 |
|  | Obesity/overweight | 1,685,237 | 5,591,976,083 | 110.1 | 109.9 | 142.2 |
|  | Previous SSTI | 1,607,555 | 2,468,083,750 | 237.9 | 237.5 | 238.3 |
|  | HIV/AIDS | 28,649 | 99,673,243 | 105.0 | 103.8 | 106.2 |
|  | Chronic kidney disease | 1,315,930 | 2,970,818,396 | 161.8 | 161.5 | 162.1 |
|  | Malignant neoplasm | 826,521 | 2,292,468,893 | 131.7 | 131.4 | 132.0 |
|  | Peripheral artery and arteriovenous disease | 1,915,096 | 3,878,673,878 | 180.3 | 180.1 | 180.6 |
|  | Chronic liver disease | 754,123 | 2,372,210,492 | 116.1 | 115.9 | 116.4 |

CI: confidence interval; dis.: disease; HIV: human immunodeficiency virus; LL/UL: lower limit/upper limit; N: number.

* Patients with one of the listed comorbidities could also have any of the other 8 comorbidities. e.g. Diabetes only or Diabetes associated with one or several other comorbidities (e.g., Dermatitis/eczema).

Table S2 Average cost per skin and soft tissue infection (SSTI) episode by year, age group and type of SSTI - All episodes* (outpatient, emergency room and inpatient)

| **Analysis** | **Subgroup** | **No. episodes** | **Mean** | **SD** | **95% CI** | | **Median** | **Q1** | **Q3** |
| --- | --- | --- | --- | --- | --- | --- | --- | --- | --- |
|  |  |  |  |  | **LL** | **UL** |  |  |  |
| Overall |  | 5,894,894 | 3333.9 | 20,029.7 | 3317.8 | 3350.1 | 189.7 | 95.6 | 509.6 |
| By year | 2010 | 499,677 | 2255.2 | 17,448.3 | 2206.8 | 2303.5 | 173.3 | 91.8 | 429.2 |
|  | 2011 | 496,656 | 2136.0 | 15,326.4 | 2093.3 | 2178.6 | 170.3 | 91.8 | 422.0 |
|  | 2012 | 514,640 | 2314.1 | 18,734.8 | 2262.9 | 2365.3 | 169.7 | 91.8 | 424.1 |
|  | 2013 | 521,536 | 2541.1 | 18,111.6 | 2491.9 | 2590.3 | 181.1 | 93.5 | 442.0 |
|  | 2014 | 470,951 | 2790.1 | 22,836.3 | 2724.8 | 2855.3 | 185.0 | 94.2 | 468.2 |
|  | 2015 | 488,115 | 3085.4 | 21,467.9 | 3025.2 | 3145.6 | 187.4 | 92.9 | 501.4 |
|  | 2016 | 498,576 | 3641.8 | 20,767.2 | 3584.2 | 3699.4 | 189.6 | 93.5 | 533.3 |
|  | 2017 | 555,989 | 3968.1 | 19,544.3 | 3916.7 | 4019.5 | 193.7 | 100.2 | 602.3 |
|  | 2018 | 615,533 | 4200.2 | 20,581.9 | 4148.8 | 4251.6 | 207.4 | 102.0 | 636.6 |
|  | 2019 | 649,395 | 4424.1 | 21,499.5 | 4371.8 | 4476.4 | 207.9 | 102.0 | 665.4 |
|  | 2020 | 567,063 | 4650.0 | 22,229.5 | 4592.2 | 4707.9 | 205.7 | 99.1 | 640.3 |
| By age category (y) | 0 - <5 | 149,514 | 524.7 | 8973.0 | 479.0 | 570.3 | 120.8 | 90.6 | 197.1 |
|  | 5 - <18 | 563,353 | 476.6 | 5589.0 | 461.9 | 491.3 | 126.4 | 90.6 | 231.1 |
|  | 18 - <45 | 1,295,543 | 1374.3 | 10,624.8 | 1355.8 | 1392.7 | 179.9 | 93.9 | 409.7 |
|  | 45 - <65 | 1,521,716 | 3753.6 | 21,358.1 | 3719.3 | 3788.0 | 205.6 | 101.9 | 606.2 |
|  | ≥65 | 2,535,074 | 4923.4 | 24,800.5 | 4892.2 | 4954.6 | 216.2 | 102.8 | 753.0 |
| By sex | Male | 2,862,103 | 3693.3 | 21,589.8 | 3668.0 | 3718.7 | 199.2 | 97.8 | 550.8 |
|  | Female | 3,202,304 | 3012.2 | 18,518.4 | 2991.6 | 3032.7 | 180.5 | 92.4 | 473.9 |
|  | Unknown | 793 | 5020.1 | 18,461.2 | 3729.2 | 6311.1 | 292.2 | 126.9 | 1291.2 |
| By SSTI type** | Type 1 | 311,135 | 21,171.1 | 60,619.4 | 20,955.3 | 21,386.9 | 1630.3 | 192.6 | 20,371.7 |
|  | Type 2 | 1,010,056 | 8179.0 | 30,917.9 | 8117.4 | 8240.6 | 403.4 | 137.7 | 2338.7 |
|  | Type 3 | 3,319,106 | 1536.6 | 7473.3 | 1528.5 | 1544.8 | 199.9 | 105.0 | 467.8 |
|  | Type 4 | 1,424,903 | 248.5 | 1361.2 | 246.3 | 250.8 | 118.2 | 85.7 | 202.5 |

* excluding outpatient episodes with hospitalization with SSTI as secondary diagnosis: costs include the visits, hospitalization and associated procedures and treatments related to claims with SSTI recorded as the first diagnosis.

** SSTI Type: 1, surgical site infection & infection due to device or graft; 2, chronic ulcer of skin; 3, abscess, cellulitis & /erysipelas; 4, folliculitis, impetigo, furuncle, mastitis & other

CI: confidence interval; dis.: disease; HIV: human immunodeficiency virus; LL/UL: lower limit/upper limit; Q1/Q3: first and third quartiles; SD: standard deviation; y: years of age.

**Figure S1** Percentage of recurrent skin and soft tissue infections (SSTI) by demographic and socio-economic factors


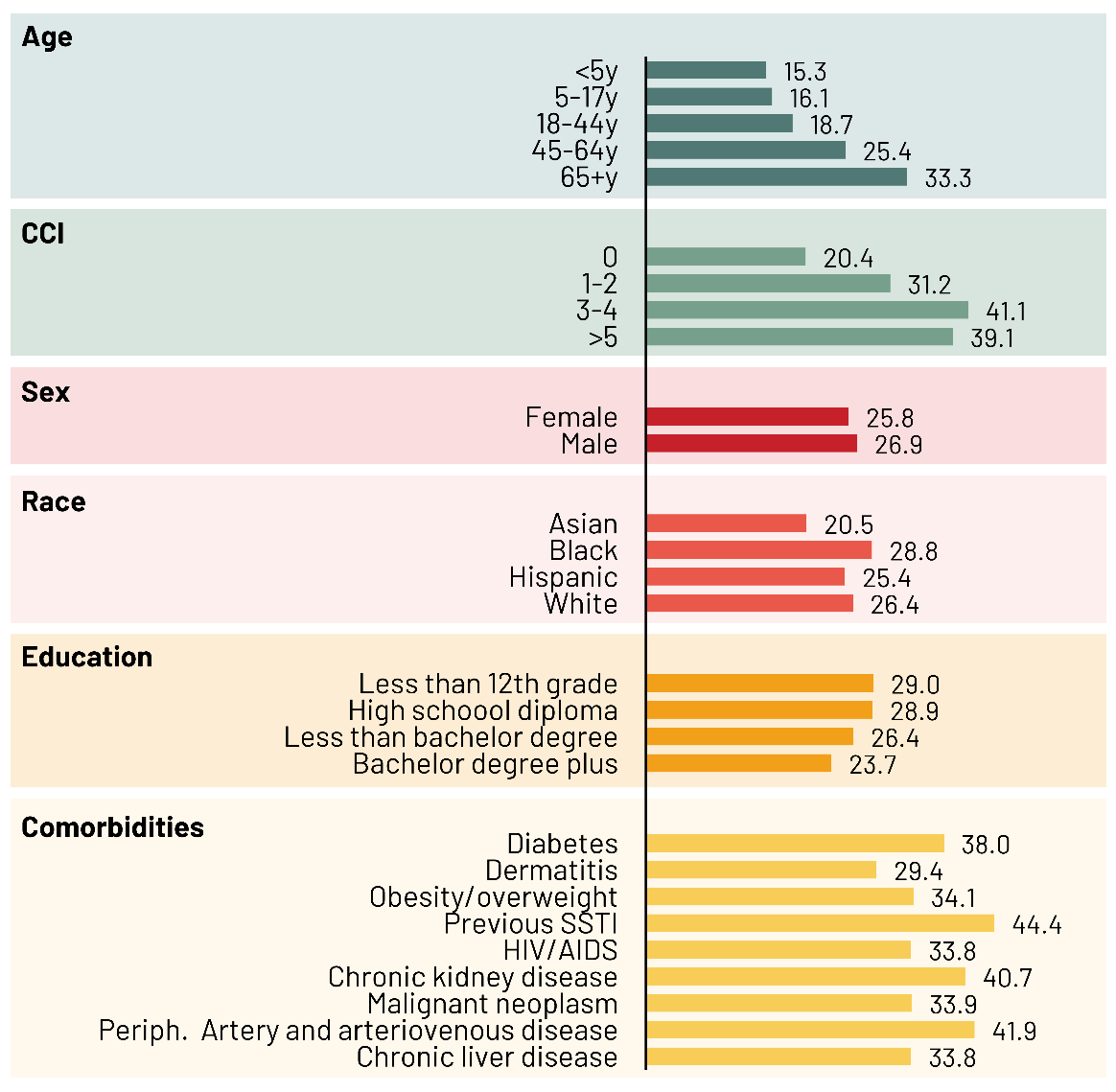


CCI: Charlson Comorbidity Index; HIV: human immunodeficiency virus; SSTI: skin and soft tissue infections.

**Figure S2** Mean and median outpatient costs (US dollars) skin and soft tissue infections (SSTI) by age, sex and type of SSTI


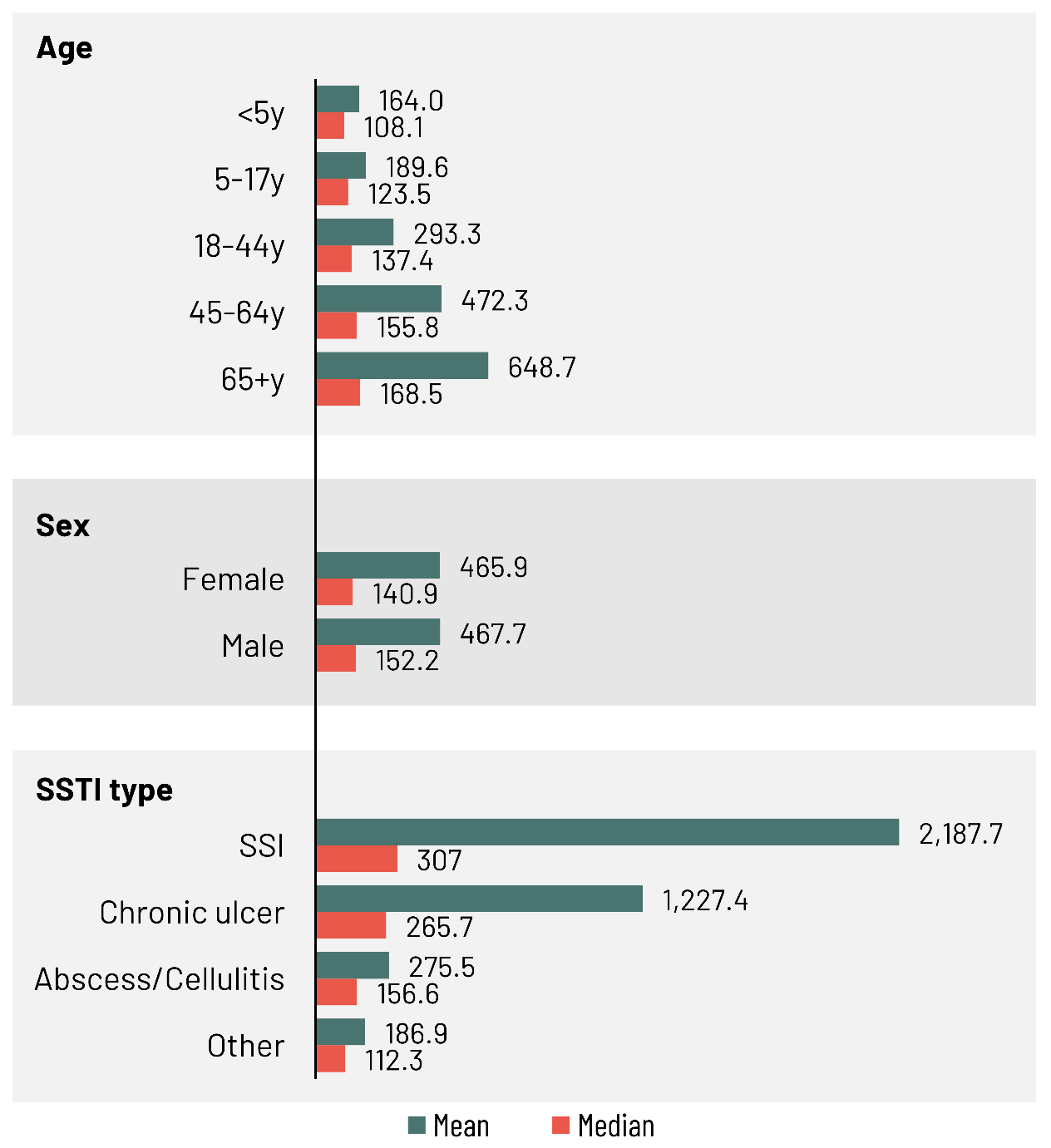


HIV: human immunodeficiency virus; Other: folliculitis, impetigo, furuncle, mastitis & other; SSI: surgical site infection & infection due to device or graft; SSTI: skin and soft tissue infections; y: years of age.
