# Supplementary material for: The Incidence of Skin and Soft Tissue Infections in the United States and Associated Healthcare Utilization Between 2010 and 2020
Source: Open Forum Infect Dis. 2024 May 7;11(6):ofae267. doi: 10.1093/ofid/ofae267 (PMC11146672; doi:10.1093/ofid/ofae267)
Supplement: ofae267_Supplementary_Data [file ofae267_supplementary_data.zip › ofae267_Plain language summary_Supplement.docx]

## Plain Language Statement

### What is the Context?

- Skin and soft tissue infections (SSTI) are very common, and studies conducted previously in the US showed that the incidence of these infections was increasing. These data are up to a decade old, and it is not known if the rate of SSTI is still increasing in the US.

### What is new?

- Our study evaluates the rate of SSTI in the US, which appears to have stabilized. However, the SSTI profile has been changing, with more patients developing chronic ulcers than before, leading to higher hospitalizations and mortality than previously reported.
- To the best of the authors’ knowledge, this is the first time that the incidence of SSTI has been measured among patients with a single specified comorbidity (eg, diabetes only) and those with multiple comorbidities (eg, diabetes and chronic kidney disease), enabling to isolate the attributable incidence of SSTI according to specific comorbidities.

### What is the Impact?

- This study provides up-to-date information on the importance of SSTI in the US, showing that the incidence of SSTI is not declining, contrary to previous assumptions.
- The study helps to identify people at risk of SSTI and to estimate the benefit from preventive interventions to reduce their risk.
