# Supplementary figures and images for: The Incidence of Skin and Soft Tissue Infections in the United States and Associated Healthcare Utilization Between 2010 and 2020
Source: Open Forum Infect Dis. 2024 May 7;11(6):ofae267. doi: 10.1093/ofid/ofae267 (PMC11146672; doi:10.1093/ofid/ofae267)

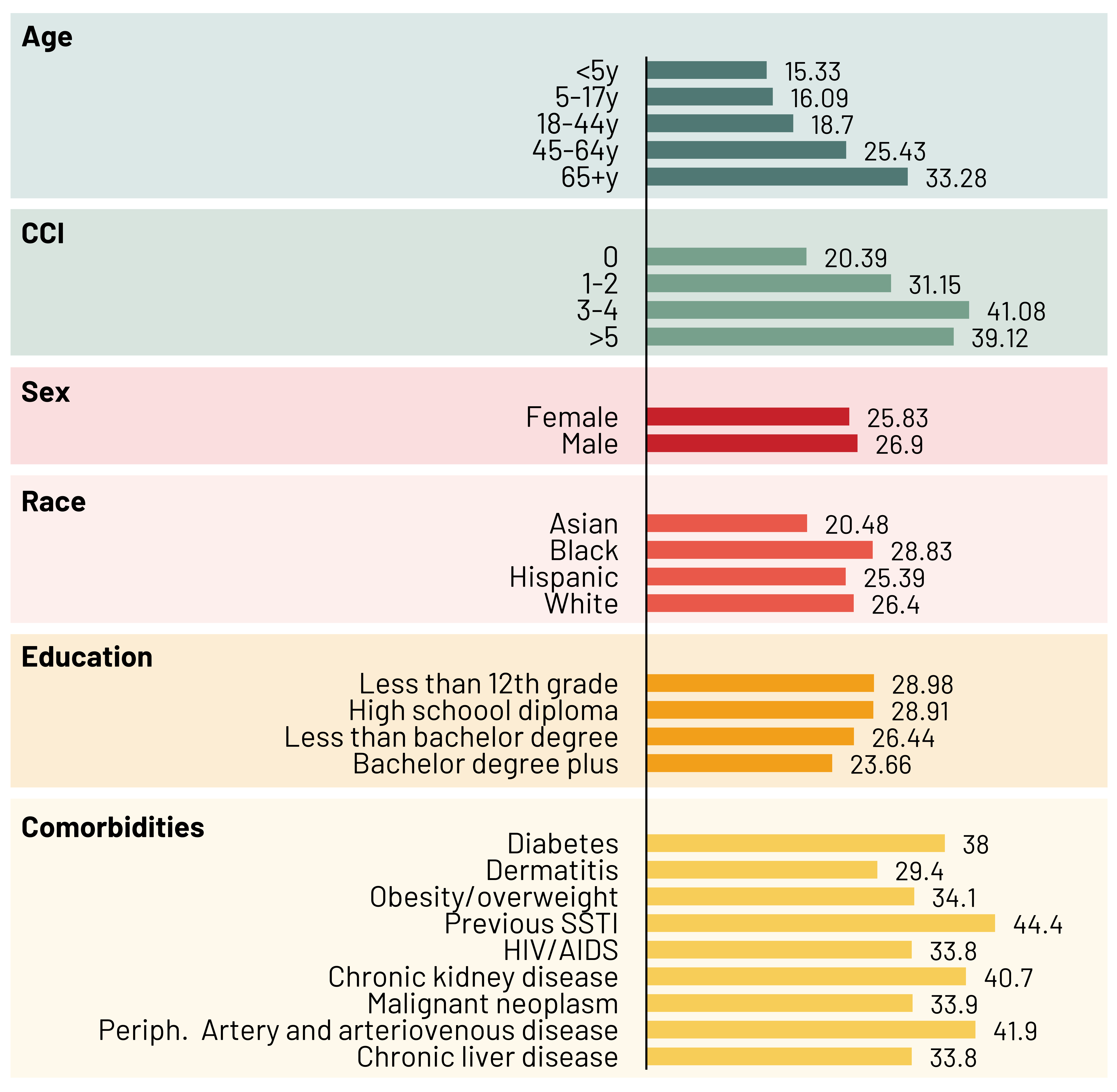

Supplement: ofae267_Supplementary_Data [file ofae267_supplementary_data.zip › EPI_OPTUM db study_Figure S1.tif]

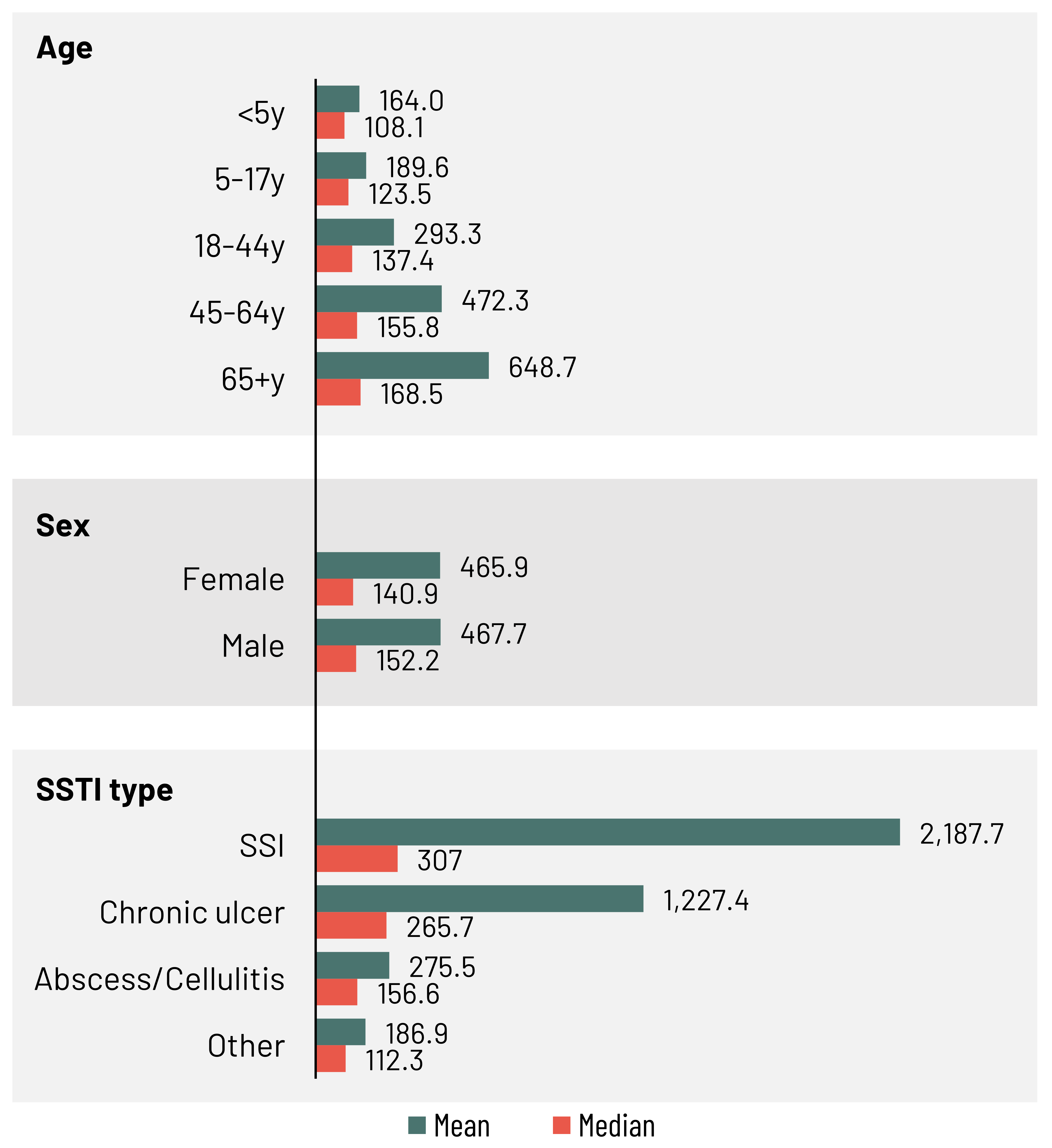

Supplement: ofae267_Supplementary_Data [file ofae267_supplementary_data.zip › EPI_OPTUM db study_Figure S2.tif]
